# Effect of TiO_2_ and Al_2_O_3_ Addition on the Performance of Chitosan/Phosphotungstic Composite Membranes for Direct Methanol Fuel Cells

**DOI:** 10.3390/membranes13020210

**Published:** 2023-02-08

**Authors:** Andrea Zaffora, Elena Giordano, Valentina Maria Volanti, Leonardo Iannucci, Sabrina Grassini, Irene Gatto, Monica Santamaria

**Affiliations:** 1 Dipartimento di Ingegneria, Università degli Studi di Palermo, Viale delle Scienze, Ed. 6, 90128 Palermo, Italy; 2 Dipartimento di Scienza Applicata e Tecnologia, Politecnico di Torino, Corso Duca degli Abruzzi 24, 10129 Torino, Italy; 3 Istituto di Tecnologie Avanzate per l’Energia “Nicola Giordano”(ITAE), Consiglio Nazionale delle Ricerche (CNR), Via Salita S. Lucia sopra Contesse 5, 98126 Messina, Italy

**Keywords:** chitosan, phosphotungstic acid, TiO_2_, Al_2_O_3_, inorganic filler, DMFC, methanol permeability, hybrid membranes, proton exchange membrane, power density

## Abstract

Composite chitosan/phosphotungstic acid (CS/PTA) with the addition of TiO_2_ and Al_2_O_3_ particles were synthesized to be used as proton exchange membranes in direct methanol fuel cells (DMFCs). The influence of fillers was assessed through X-ray diffraction, scanning electron microscopy, thermogravimetric analysis, liquid uptake, ion exchange capacity and methanol permeability measurements. The addition of TiO_2_ particles into proton exchange membranes led to an increase in crystallinity and a decrease in liquid uptake and methanol permeability with respect to pristine CS/PTA membranes, whilst the effect of the introduction of Al_2_O_3_ particles on the characteristics of membranes is almost the opposite. Membranes were successfully tested as proton conductors in a single module DMFC of 1 cm^2^ as active area, operating at 50 °C fed with 2 M methanol aqueous solution at the anode and oxygen at the cathode. Highest performance was reached by using a membrane with TiO_2_ (5 wt.%) particles, i.e., a power density of 40 mW cm^−2^, almost doubling the performance reached by using pristine CS/PTA membrane (i.e., 24 mW cm^−2^).

## 1. Introduction

Direct methanol fuel cells (DMFCs) are among the most appealing green technologies in energy supply for portable electronic devices, due to the several advantages related to the usage of methanol aqueous solution, such as high volumetric energy density of methanol (i.e., 4820 Wh L^−1^) [1], low operating temperature, easy fuel storage and transportation and quick refueling [2]. Recently, DMFCs have been classified as the most cost-effective technology as a power source for forklift applications, considering the much lower cost of methanol infrastructure with respect to that of hydrogen and a proper improvement in performance and reduction in PGM-based catalyst loading [3]. Additionally, the military sector is integrating DMFC systems within its applications since DMFC can represent a power source that is lightweight and compact, with very well controlled fuel supply, water and heat management. Moreover, the use of DMFCs eliminate requirements for fuel reforming and/or large onboard hydrogen storage tanks, which are key issues for the usage and operation of PEMFC systems.

It is evident that DMFCs produce CO_2_ as a product of methanol oxidation reaction. To overcome this issue, DMFC systems can use CO_2_-capturing systems [4]. Furthermore, from an analysis produced by the USA Department of Energy (DoE) Hydrogen and Fuel Cells Program Record, whether the methanol is used as a source of hydrogen that is then fueled in a fuel cell electric vehicle (FCEV) or is used directly in a DMFC-based FCEV, the evaluated well-to-wheels (WTW) greenhouse gas (GHG) emissions are lower than both internal combustion vehicles and hybrid vehicles, being almost 300 g_CO2,e_ mile^−1^ [5].

However, some limitations still delay a full and widespread DMFC technology commercialization, mostly due to the sluggish methanol oxidation reaction (MOR) at the anode and severe methanol crossover through the membrane from anodic to cathodic compartment [6,7]. The sluggish kinetic of MOR is basically due to the complex reaction mechanism that involves many steps to succeed in exchanging six moles of electrons per mole of methanol, according to the reaction:

(1) $${\mathrm{CH}}_{3}\mathrm{OH}\;+\;H_{2}O\;\to {\mathrm{CO}}_{2}+6H^{+}+6e^{-}$$

Typical Pt/C electrocatalysts cannot be used for MOR since strong adsorption of carbon monoxide (CO) causes active site poisoning. For this reason, incorporation of another metal in anodic electrocatalyst to form bimetallic compounds is the common strategy to reduce activation overvoltage of MOR, because the second element (e.g., Ru, Pd, Cu, Rh or Co) provides hydroxyl species helping the oxidation of CO to CO_2_ [2,8,9,10]. To increase DMFC efficiency, high catalyst loadings should be used, but this would increase the overall cost of the device. On the other hand, decreasing catalysts loadings can lead to lower cost, which is important for a widespread distribution of the technology, but it could also lead to a lower catalyst durability.

Methanol crossover from anode to cathode is the other phenomenon that can lead to huge voltage losses, reducing the overall efficiency of the device. In fact, methanol diffuses through the polymer electrolyte membrane because of the concentration gradient and because of the electroosmotic drag due to the proton migration from anodic to cathodic compartment [11,12]. This causes cathode depolarization with consequent high difference between the electromotive force value and the open circuit voltage (OCV) value, possible cathodic electrocatalyst poisoning (that is typically Pt-based) and consumption of O_2_ [7,13].

Current strategies to reduce methanol crossover enhancing DMFC efficiency are based on the modification of the polymer electrolyte membrane as a barrier to the fuel diffusion, but without compromising proton conductivity and chemical stability, which are necessary for high-performance fuel cells. The introduction of filler nanoparticles can make methanol diffusion difficult because of membrane channel blocking inducing steric hindrance for methanol crossover and/or because of an increase in channels’ tortuosity which, unfortunately, is often accompanied by a decrease in proton conductivity [13]. These strategies are usually adapted to decrease methanol permeability of Nafion^®^ proton exchange membranes that are the state-of-art membranes for DMFCs. Nafion^®^ and Nafion^®^-based membranes, despite exhibiting high proton conductivity and durability, suffer from high methanol permeability and high manufacturing costs [14,15], following a non-environmentally friendly production route.

As possible substitutes of Nafion^®^-based membranes for DMFCs, biopolymer-based membranes are considered due to their environmental friendliness. In particular, chitosan (CS) is among the most used biopolymers because of its abundance, since it derives from the N-deacetylation of chitin, its cost-effectiveness and its biocompatibility. CS-based membranes are then used in various applications, such as electrodialysis, ultra and nanofiltration [16,17,18]. Despite their pros, CS-based membranes do not have high enough proton conductivity to be used in FCs as solid electrolytes. In this frame, heteropolyacids (HPAs) can be used to increase CS-based membranes’ proton conductivity. In fact, HPAs suffer from leaching when used as solid electrolytes in FCs’ applications, leading to poor durability. Phosphotungstic acid (PTA) is one of the HPAs that were successfully employed to form composite CS/HPA membranes to be used as proton exchange membranes in hydrogen-fed FCs [19,20,21,22,23] and in DMFCs. In particular, CS/PTA membranes have been successfully tested as proton exchange membranes, reaching high performances at 70 °C with relatively low catalyst loadings at both anode and cathode [24]. Nevertheless, they showed quite high methanol permeability values, demonstrating that there is still room for improving their performances and decreasing methanol crossover phenomenon.

In order to further enhance DMFC performance employing CS/PTA proton exchange membranes, trying to use materials that can be sustainable in every production phase and in their final disposal, in this work we synthesized hybrid inorganic-organic CS/PTA proton exchange membranes [25] with the addition of TiO_2_ and Al_2_O_3_ particles to decrease methanol permeability but retain high proton conductivity and durability. Membranes were characterized with X-ray diffraction and scanning electron microscope to gain information about crystallinity and morphology of the membranes as a function of the fillers’ nature. Membrane properties such as liquid uptake, ion exchange capacity and methanol permeability were studied. Finally, composite membranes were tested in a single DMFC module fed with methanol and oxygen.

## 2. Materials and Methods

### 2.1. Materials

Chitosan powder, acetic acid, phosphotungstic acid (H_3_PW_12_O_40_ × H_2_O) and Al_2_O_3_ powders were provided by Sigma Aldrich. TiO_2_ powder was supplied by Carlo Erba.

### 2.2. Membrane Synthesis

Membranes were synthesized by ionotropic gelation process on an anodic alumina membrane (AAM) employed as porous support that was previously impregnated by PTA, as described elsewhere [19,23]. CS solution was prepared by mixing CS powder (2% *w*/*v*), acetic acid (2% *w*/*v*) and distilled water to obtain protonation and solubilization of CS.

To add fillers into the membranes, inorganic powders (2% *w*/*v* and 5% *w*/*v*) were added to the aqueous acetic acid solution. This mixture was subjected to an ultrasonic cycle of 20 min so that the powder could be homogeneously dispersed in the aqueous solution. Finally, CS powder was added to the aqueous solution with acetic acid and mixed. Solutions were stirred for, at least, 24 h before use. Higher content of inorganic nanoparticles led to worse performances or non-uniform dispersion in acetic acid aqueous solution.

**Figure 1:**
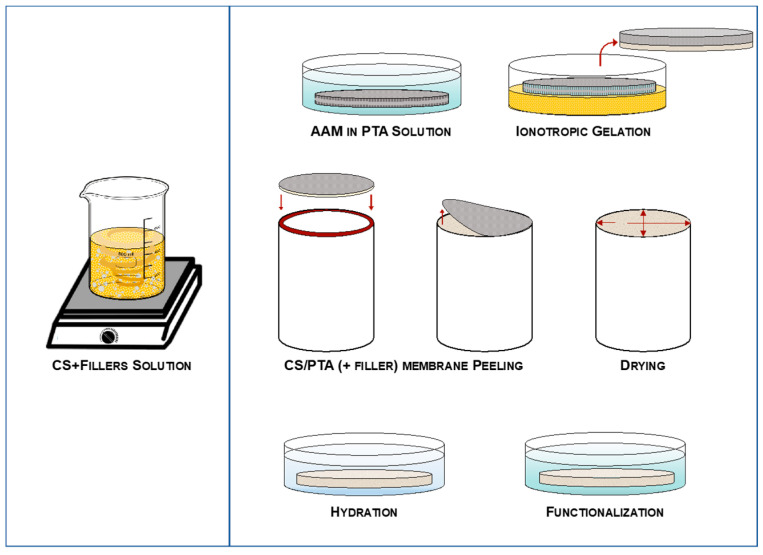
Ionotropic gelation process of the hybrid CS/PTA + filler membranes.

CS solutions and fillers were put in contact with a porous medium previously impregnated with an aqueous PTA solution (0.38 M) to induce membrane reticulation (cross-linking). The reticulation time (60 min in this work) was useful for controlling the thickness of the membrane. Finally, all the membranes were put in the aqueous PTA solution for 24 h for the functionalization process, i.e., to increase the proton conductivity of the membranes. The whole synthesis process is schematically reported in Figure 1.

### 2.3. X-ray Diffraction (XRD)

X-ray diffraction (XRD) patterns were collected using a Pan Analytical Empyrean, powder X-ray diffractometer with a copper anode (Cu Kα radiation, λ = 0.15405 nm, 30 kV, 30 mA). XRD patterns were recorded over the 2θ angle range from 10° to 90° with a step size of 0.03° and a scan speed of 4° min^−1^.

### 2.4. SEM Characterization

Scanning electron microscope (SEM) analysis was conducted using a Philips XL30 ESEM coupled with EDX equipment. Prior to image acquisition, several pieces of each sample were fixed on the metal stubs with Ag conductive paste.

### 2.5. Liquid Uptake

Liquid uptake was calculated through the following relationship [26]:

(2) $$L.U.\;\left[\%\right]=\frac{W_{wet}-W_{dry}}{W_{dry}}\times 100$$

where *W_dry_* (mg) is the weight of the dried membrane and *W_wet_* (mg) is the weight of the wet membrane. Specifically, once synthesized, the membrane was washed for 5 min in distilled water to remove any traces of membrane synthesis solution and then dried for, at least, 24 h at 25 °C. After the membrane was weighed, it was immersed for 24 h in methanol aqueous solution at different concentrations (1 M, 2 M and 5 M). The membrane was weighed by removing excess solution to estimate the correct *W_wet_* value. Each liquid uptake measurement was repeated four times for all characterized membranes.

### 2.6. Ion Exchange Capacity

The ion exchange capacity (IEC) of the membranes was determined using a titration process. IEC was measured for functionalized membranes.

Membrane weight (*W_dry_*) after a drying step was measured, then it was washed and left for 15 min in distilled water to remove excess acid deposited on the surface. The membrane thus was immersed in a 1 M NaCl solution for 2 h so that the exchange between H^+^ contained in the membrane with the Na^+^ ions in the solution takes place. Therefore, the solution had an acidic character due to the formation of HCl, so it was neutralized by the addition of a 0.01 M NaOH solution. The volume of NaOH (*V_NaOH_*, [L]) required to reach the equivalent point (pH = 7) was calculated. The *IEC* of each membrane was, then, determined using the following equation [26]:

(3) $$IEC=\frac{\left[NaOH\right]\;V_{NaOH}}{W_{dry}}$$

### 2.7. Methanol Permeability

The methanol permeability of the synthesized membranes was estimated using a two-compartment diffusion cell at 70 °C.

The membrane was placed between the donor compartment (A), where a 1 M methanol aqueous solution was present, and the receptor compartment (B), where distilled water was present. At first, the two compartments were loaded with 96 mL of deionized water each; when the two compartments reached 70 °C, 4 mL of water was added to B and 4 mL of methanol to A.

Once the test began, liquid samples were taken from the downstream cell (B compartment) at different time intervals, specifically after 1 min, 10 min, 30 min, 1 h, 3 h, 5 h and 7 h from the start of the test, in order to evaluate the trend of methanol concentration as a function of time (C_B_(t)). The samples taken were then analyzed with a GC-2010 SHIMADZU gas chromatograph, inside which 0.2 μL of solution was injected for each measurement via a 2 μL HAMILTON syringe.

The methanol permeability of the membrane was calculated by the following equation [27]:

(4) $$P=\frac{1}{C_{A}}\;\left(\frac{\Delta C_{B}}{\Delta t}\right)\left(\frac{L\;V}{A}\right)$$

where *P* is the methanol diffusion permeability into the membrane (cm^2^ s^−1^), *C_A_* is the concentration of methanol in cell *A* (mol L^−1^), Δ*C_B_(t)*/*Δt* is the slope of the change in the molar concentration of methanol in the *B* cell as a function of time (mol L^−1^ s^−1^), *V* is the volume of each diffusion tank (cm^3^), *L* is the membrane thickness (cm) and *A* is the membrane area (cm^2^).

### 2.8. Thermogravimetric Analysis

Thermogravimetric analysis (TGA) was carried out to study the thermal stability of hybrid inorganic-organic membranes by using a thermobalance TG/DTA NETZSCH 449 F1 Jupiter. Membrane samples were placed in an alumina crucible and heated from 30 to 400 °C with a heating rate of 10 °C min^−1^ in nitrogen atmosphere.

### 2.9. Single DMFC Performances

The single cell characterizations were carried out by using in-house prepared electrodes. For the anode a 60% Pt-Ru/C (Alfa Aesar) electrocatalyst with a Pt loading of 2.3 mg cm^−2^ was used, while a 40% Pt/C (Alfa Aesar) with a Pt loading 0.5 mg cm^−2^ was used at the cathode. The catalytic inks, obtained by mixing the electrocatalysts with Nafion (5 wt% hydro-alcoholic solution IonPower-LQ1105) and a pore-former (ammonium carbonate), were deposited by spray coating technique onto a commercial gas diffusion layer Sigracet-24 BC (from the SGL group), as described elsewhere [28,29].

Membrane electrode assembly (MEA) was obtained by placing electrodes and membrane in the single cell module with an applied torque of 4 Nm for each measurement. To evaluate the membranes performance in the DMFC, a 2 M methanol aqueous solution was used, fed to the anode at a flow rate of 3 mL min^−1^, while humidified oxygen (99.5% purity) was fed to the cathode at 50 mL min^−1^. Oxygen and methanol were fed using high-purity graphite plates with high electrical and thermal conductivity. In addition, silicone gaskets were inserted between the MEA and the graphite plates. Gold-plated plates were used as current collectors, which in turn were joined to the graphite plates. Electrochem Inc. DMFC station was used for the DMFC in-cell characterization. EIS measurements were made by superimposing a 10 mV amplitude sinusoidal signal in the frequency range of 100 kHz to 10 mHz on the constant continuous cell voltage. Working and sense electrodes were connected to the cathode (oxygen side) whilst counter and reference electrodes were connected to the anode (methanol side). ZSimpWin software was used to fit the obtained EIS spectra. Electrical equivalent circuit used for the fitting procedure is discussed in Section 3.6. All cell tests were performed at 50 °C with a Parstat 4000 (Princeton Applied Research) and refer to an active (apparent) area of 1 cm^2^. A triple serpentine flow field was used to supply reactants to the cell.

## 3. Results and Discussion

### 3.1. XRD Analysis

XRD analysis was performed to study the crystallinity of the membranes and verify the correct incorporation of the fillers within the chitosan/PTA matrix. In the case of proton exchange membranes used in DMFC, crystallinity degree is important since it affects the mass transport across the membrane [30,31], i.e., methanol crossover (in the case of DMFC) with consequent performance decrease. XRD pattern for CS/PTA membrane without any filler is reported in Figure S1. Reflection at 2θ = 20.5° is due to the presence of Form II polymorph of chitosan. This reflection is present only after the functionalization step [20], which leads to an increase in crystallinity degree of the membranes.

**Figure 2:**
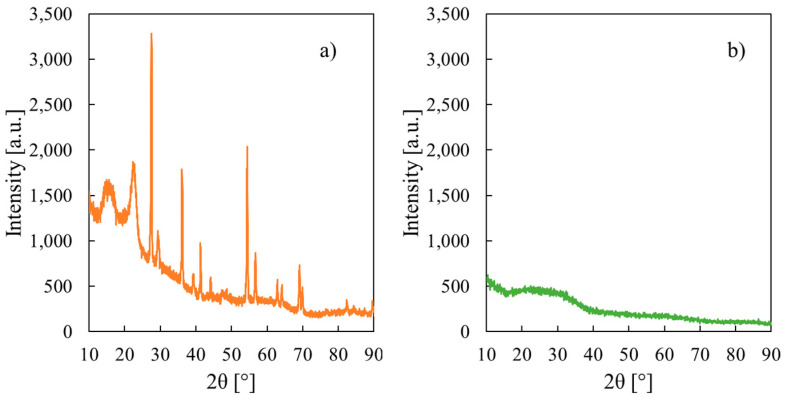
X-ray diffraction patterns related to CS/PTA membrane with (**a**) TiO_2_ and (**b**) Al_2_O_3_ particles.

XRD patterns of CS/PTA membranes after fillers addition are shown in Figure 2a,b.

With the addition of the TiO_2_ particles, the crystallinity degree of the obtained composite membrane increases if compared to pristine CS/PTA polymer (see Figure 2a), due to the inclusion of the filler particles. Characteristic reflections of rutile polymorph of TiO_2_ can be seen at 2θ = 27.6°, 36.2°, 41.4°, 54.4°, 62.8° and 69.2° [32]; therefore, TiO_2_ particles were successfully incorporated into the CS/PTA membranes synthesized by ionotropic gelation process.

The effect on the crystallinity degree of addition of Al_2_O_3_ particles during the synthesis of the membranes is completely different with respect to TiO_2_ particles, as can be noted in Figure 2b where the XRD pattern for membrane with Al_2_O_3_ particles is reported. Diffraction peaks relative to pristine CS/PTA membranes, which can be identified from Figure S1, are flattened after the inclusion of Al_2_O_3_ particles. Furthermore, diffraction peaks of Al_2_O_3_ powder (see Figure S2) that was used for preparing the Al_2_O_3_-containing membranes are not present in XRD pattern shown in Figure 2b. These findings suggest an increase in the amorphous degree of the membranes and the incorporation of a small concentration of Al_2_O_3_ particles.

### 3.2. Membranes Morphology and TGA Analysis

CS/PTA hybrid inorganic-organic membranes were morphologically characterized at the microscale by SEM. In previous works, it has been demonstrated that PEMs for hydrogen-fed and methanol-fed low-T fuel cells can be successfully synthesized through ionotropic gelation method [21,22,24]. These PEMs were compact and flaw-free, without the presence of micro/macro voids that can negatively affect cell performance. The addition of inorganic fillers can lead to changes to the structural and morphological properties of PEMs.

**Figure 3:**
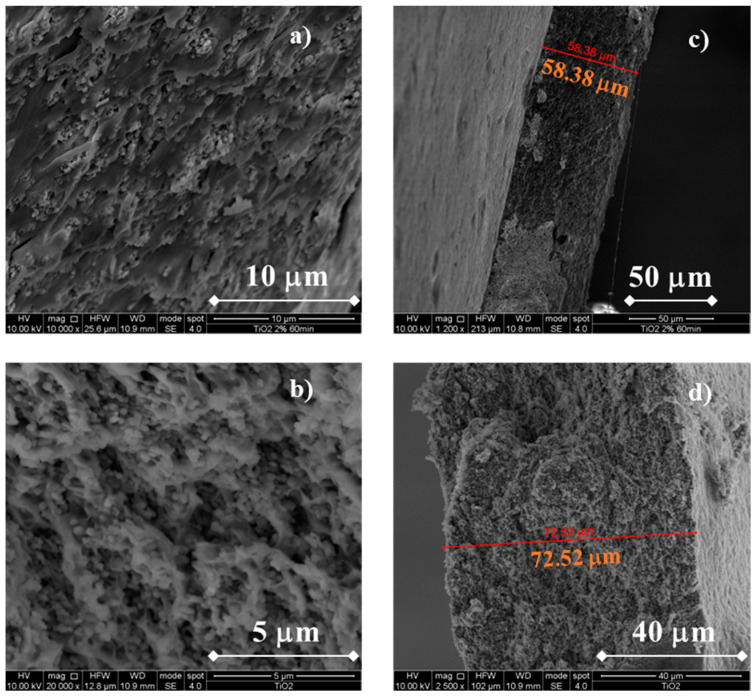
SEM cross-sectional micrograph of a CS/PTA membrane with TiO_2_. Morphology of the (**a**) CS/PTA +TiO_2_ (2% wt) membrane, (**b**) CS/PTA + TiO_2_ (5% wt) membrane and cross section of the (**c**) CS/PTA + TiO_2_ (2% wt) membrane and (**d**) CS/PTA + TiO_2_ (5% wt) membrane.

In Figure 3, SEM micrographs related to the morphology of TiO_2_-containing membranes are reported.

Notably, the addition of TiO_2_ particles does not cause any significant changes to the membranes, at least from a morphological point of view. In fact, compact and uniform membranes can be appreciated in Figure 3a,c, regardless of the TiO_2_ content added during the membranes synthesis. TiO_2_ particles were uniformly dispersed across the entire thickness without sign of particle aggregates/clusters. It is noteworthy to mention that a higher TiO_2_ wt% concentration during the synthesis leads to thicker membranes. In fact, using TiO_2_ 2 wt%, membrane thickness is ~58 μm whilst using a TiO_2_ 5 wt% concentration the membrane thickness is ~73 μm. These thickness values are lower than the usual Nafion membranes thickness used for DMFC (~125 μm for Nafion^®^ 115) and are in the same order of magnitude of pristine CS/PTA membranes without the addition of any filler, indicating the optimal dispersion of the particles inside the membranes.

**Figure 4:**
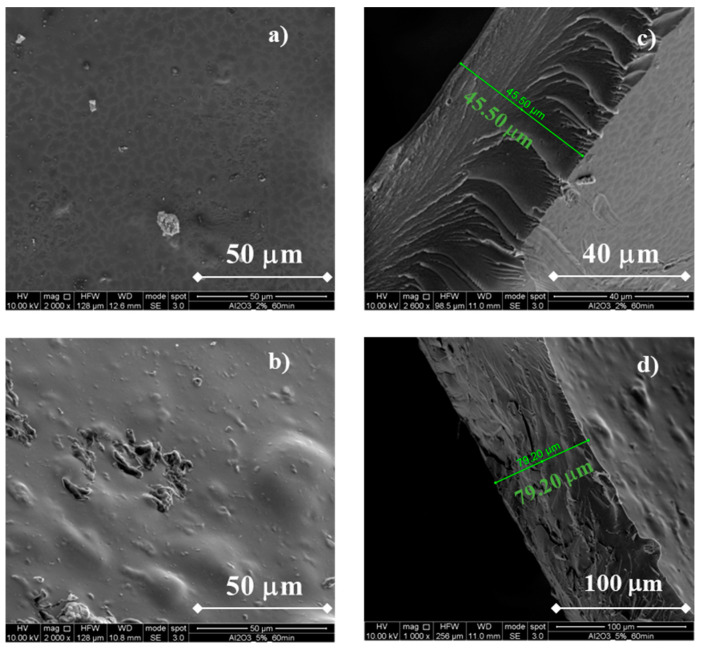
SEM cross-sectional micrograph of a CS/PTA membrane with Al_2_O_3_. Morphology of the (**a**) CS/PTA + Al_2_O_3_ (2% wt) membrane, (**b**) CS/PTA + Al_2_O_3_ (5% wt) membrane and cross section of the (**c**) CS/PTA + Al_2_O_3_ (2% wt) membrane and (**d**) CS/PTA + Al_2_O_3_ (5% wt) membrane.

In Figure 4, SEM micrographs related to the morphology of Al_2_O_3_-containing membranes are shown.

It can be noted that the membranes still appear without any flaws, confirming that the addition of fillers can be successfully performed during the ionotropic gelation process. Using Al_2_O_3_ 2 wt%, membrane thickness is ~46 µm, i.e., slightly lower than that obtained using TiO_2_ 2 wt%, whilst using a Al_2_O_3_ 5 wt% concentration the membrane thickness is ~79 µm, i.e., slightly higher than that obtained using TiO_2_ 5 wt%. It is worth noting that membrane morphology (see cross sections in Figure 4b,d) is different with respect to that of TiO_2_-containing membranes. It is similar to pristine membrane morphology [20] due to a low degree of particle incorporation, leading also to amorphous membranes (see before). These different morphologies can lead to different properties, specifically crystallinity and methanol permeability (see below).

**Figure 5:**
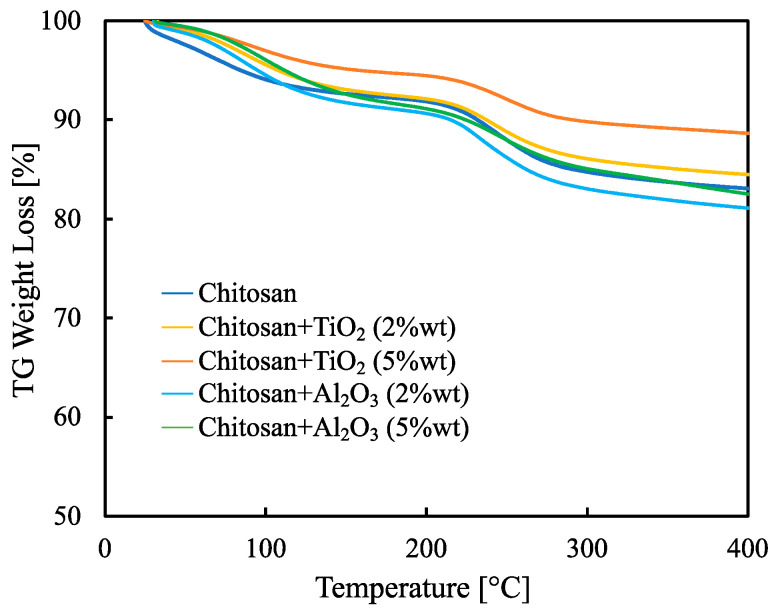
TG weight loss evaluated between 30 °C and 400 °C for all the synthesized membranes.

Weight loss, evaluated by thermo-gravimetric analysis, for all the synthesized membranes is reported in Figure 5.

Up to 100 °C, the weight loss is ≈5% and is due to the water evaporation. However, this low weight loss indicates that synthesized hybrid membranes can safely work up to 80 °C. It is worth noting that the total weight loss, evaluated at 400 °C, is only about 20% for the membrane with the worst thermal behavior, i.e., pristine CS/PTA. The addition of inorganic filler improves, in any case, the thermal stability. The hybrid inorganic-organic membrane with the lowest weight loss is the TiO_2_-containing membrane with 5% wt.

### 3.3. Liquid Uptake

Measurement of the liquid uptake of synthesized membranes is important to predict performance in single cell setup because it strongly depends on the liquid (i.e., water and methanol) content that the membrane is able to absorb and retain. In fact, one of the main issues affecting DMFC cells performance is the methanol crossover through the polymer membrane, which can permeate along with water from the anodic to cathodic compartment.

**Figure 6:**
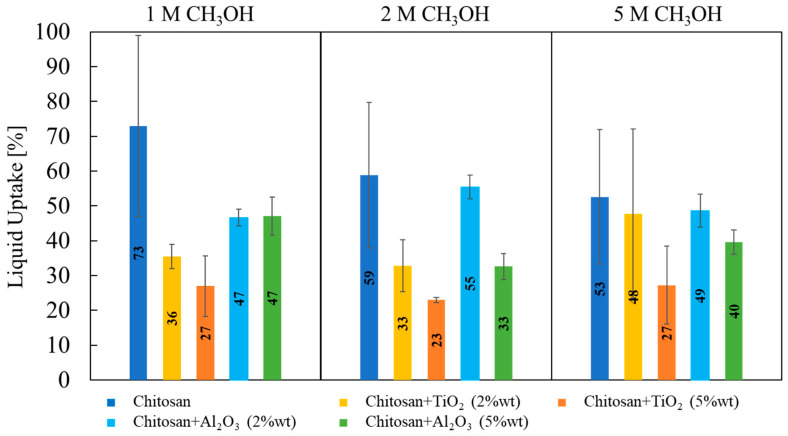
Liquid uptake values, estimated according to Equation (2), as a function of properties of synthesized membranes (filler) and methanol aqueous solution concentration.

Liquid uptake measurements were performed by the double-weighted gravimetric technique, using an aqueous solution with several methanol concentrations (i.e., 1 M, 2 M and 5 M) to assess if feed methanol concentration can affect the liquid uptake. Liquid uptake values related to different membranes and different methanol concentration are shown in Figure 6.

No significant changes in membranes liquid uptake were detected depending on the methanol concentration used for the test, meaning that these membranes do not show any tendency to retain more methanol than water.

Evaluating the effect of the nature of fillers, membranes prepared with 5% wt TiO_2_ particles have the lowest values of liquid uptake (≈23%) at any CH_3_OH concentration. On average, TiO_2_-containing membranes present lower liquid uptake than Al_2_O_3_-containing membranes, which is lower than that related to pristine CS/PTA membranes.

It is documented in the literature that water retention properties strongly depend on the surface properties of inorganic filler particles [33,34,35,36,37], considering that the strength of water adsorption on the particles is related to the charge of surface functional groups and to the particle surface area. Surface charge can be directly correlated to isoelectric point or zero charge pH, pH_pzc_, of the inorganic filler particles [38]. In particular, if the solution pH is lower than the particle pH_pzc_, the surface will be positively charged. Conversely, if the solution pH is higher than the particle pH_pzc_, the surface will be negatively charged. Furthermore, the difference between pH and pH_pzc_ can be related to the density of positive/negative site of the particles. If we consider that TiO_2_ pH_pzc_ is 5 and Al_2_O_3_ pH_pzc_ is 9.5 [38], the difference between pH and pH_pzc_ of alumina particles is higher with respect to that related to titania particles; therefore, the surface charge is higher in the case of alumina particles as filler. This causes a higher liquid uptake in the case of alumina as filler, concluding that membrane with TiO_2_ 5% wt retains less water, but at the same time it also absorbs less methanol.

### 3.4. Ion Exchange Capacity

IEC values are strictly related to the content of functional groups of the membranes, and thus are also strictly related to the membranes’ proton conductivity. To achieve higher proton conductivity, high IEC values are required.

**Figure 7:**
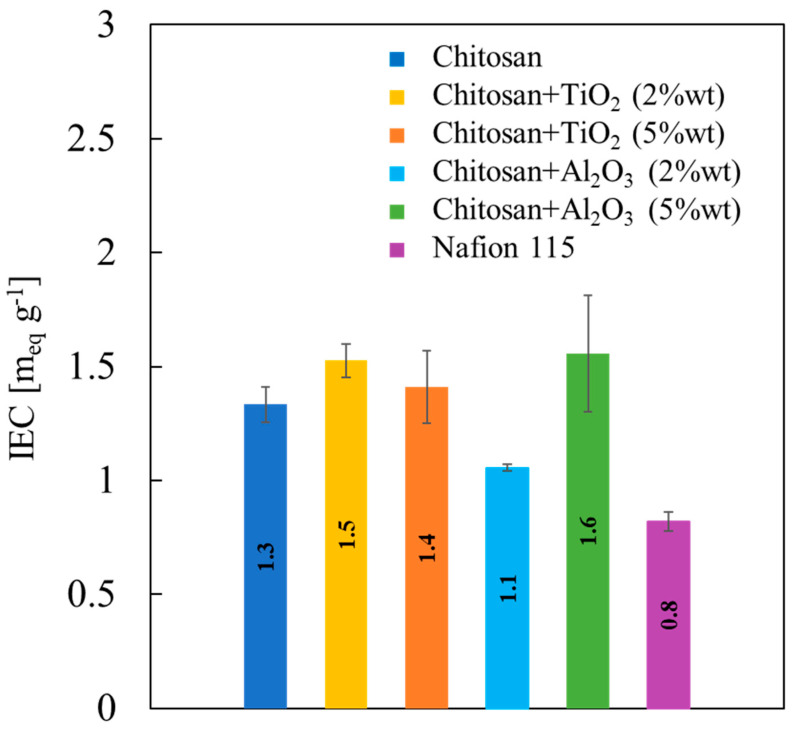
Ion exchange capacity values, estimated according to Equation (3), as a function of properties of all the synthesized membranes.

IEC values related to all the membranes studied in this work are reported in Figure 7.

As average trend, it can be noted that the addition of inorganic filler increases IEC value of the pristine CS/PTA membrane, that is, 1.3 meq g^−1^, to values close to 1.5 meq g^−1^. Only membranes with Al_2_O_3_ 2% wt present slightly lower IEC values than pristine membranes (1.1 meq g^−1^), but even higher than those measured for Nafion^®^ 115 membrane, i.e., 0.8 meq g^−1^. Therefore, adding fillers to the CS/PTA slightly enhances IEC of the pristine CS/PTA.

### 3.5. Methanol Permeability

Membranes methanol permeability is one of the most important characteristics of the PEM to be used in DMFC since methanol crossover, from anodic to cathodic compartment, can lead to dramatic performance losses. Fillers’ inclusion in PEM can be a solution to decrease methanol permeability, since their presence inside the membrane channels can be a physical obstacle to the methanol diffusion introducing tortuous pathways and/or steric blocking.

**Figure 8:**
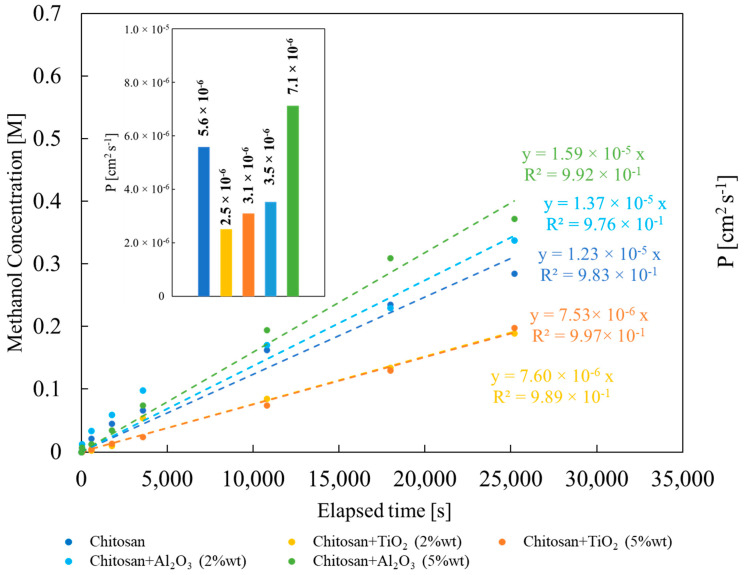
Methanol concentration dependence on time during methanol permeability measurements. Inset: methanol permeability values estimated according to Equation (4).

In Figure 8, methanol concentration in the permeate compartment (see experimental section) vs. time graph is reported for all synthesized membranes.

As expected, methanol concentration increases almost linearly with measurement time. The slope of the graph depends on the properties of the membrane, i.e., on the nature and concentration of the filler. From the slope, according to Equation (4), it is possible to estimate methanol permeability for all the studied membranes. Lowest methanol permeability value has been estimated for membrane prepared with TiO_2_ 2% wt, 2.5 × 10^−6^ cm^2^ s^−1^, whilst the highest one for the membrane prepared with Al_2_O_3_ 5% wt, 7.1 × 10^−6^ cm^2^ s^−1^ (see Figure 8). Except for the latter case, methanol permeability turned out to be lower with respect to that estimated for pristine CS/PTA membrane, 5.6 × 10^−6^ cm^2^ s^−1^. This result is coherent with the data discussed before: TiO_2_-containing membranes are more crystalline than pristine CS/PTA membrane with a lower liquid uptake, and therefore they show the lowest methanol permeability value. However, the presence of inorganic fillers leads to lower methanol permeability values, with high IEC and low water uptake.

### 3.6. Single DMFC Performance

**Figure 9:**
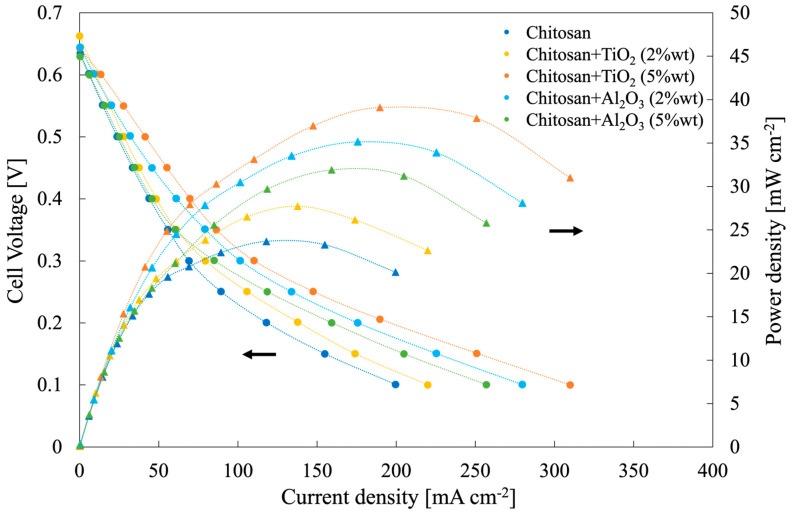
Polarization and power density curves related to a single module DMFC of 1 cm^2^ as active area, employing all the synthesized membranes, operating at 50 °C and fed with 2 M methanol aqueous solution at the anode and oxygen at the cathode.

Figure 9 shows the polarization and power density curves measured for different synthesized hybrid membranes, changing filler type and concentration, at T = 50 °C feeding 2 M methanol solution at the anode. Notably, low Pt loading (2.3 mg cm^−2^) at anode and Pt loading (0.5 mg cm^−2^) at cathode were used with respect to those reported usually in the literature [9].

Open circuit voltage (OCV) values, regardless of membrane inserted in the MEA, were lower than electromotive force, that is, 1.21 V. OCV value strongly depends on the fuel crossover phenomena, leading to a depolarization of the cathode. It is noteworthy to mention that the presence of fillers inside the membrane reduced methanol crossover with respect to pristine CS/PTA membranes due to a reduced methanol permeability. In fact, higher OCV values were measured for the membranes with fillers with respect to that prepared with Al_2_O_3_ 5% wt due to a higher methanol permeability (see Figure 8), which are comparable or higher OCV values than those obtained with Nafion membranes with the same or higher thickness values (i.e., 0.59 V—0.66 V) [39].

The highest power density value was measured using a hybrid membrane CS/PTA with TiO_2_ (5 wt%), achieving 40 mW cm^−2^, sensibly higher than that measured by using a pristine CS/PTA membrane, i.e., 24 mW cm^−2^. Hybrid membrane CS/PTA with TiO_2_ (5 wt%) was also tested with 9 h stability test (see polarization curve recorded after the test in Figure S4) demonstrating a low decrease in performance. These power density peaks are higher than that reached by using commercial Nafion^®^ 212, i.e., Nafion membrane with comparable thickness, which was 12 mW cm^−2^, mainly due to a low measured OCV value (≈0.6 V) confirming a high methanol permeability (see Figure S3). From the slope of the linear part of the polarization curves, considered as close to the membrane resistance value, *R_m_* (Ohm cm^2^), it is possible to estimate membranes’ proton conductivity, *σ* (mS cm^−1^), according to the following equation [20]:

(5) $$\sigma =\frac{L}{R_{m}A}$$

Where *A* (cm^2^) is the active (apparent area) and *L* (cm) is the membrane thickness. The highest proton conductivity values were estimated for hybrid membranes containing 5% of the filler (7.6 mS cm^−1^ and 7.3 mS cm^−1^ for TiO_2_-containing and Al_2_O_3_-containing membranes, respectively). For membranes containing 2% of the filler, 4 and 4.4 mS cm^−1^ were estimated for TiO_2_-containing and Al_2_O_3_-containing membranes, respectively.

As a general trend, all the power density values measured using membranes with the addition of filler, regardless of their nature, were higher than power density obtained by using a pristine CS/PTA membrane. In the particular case of a membrane with TiO_2_ (5 wt%) as filler, cell performance can be related to the specific features of this membrane, i.e., low liquid uptake, high IEC and low methanol permeability. By taking into account the acidic environment inside the DMFC and corresponding pH_pzc_ values, TiO_2_ and Al_2_O_3_ particles inside the solid electrolyte are negatively charged. In particular, being pH_pzc, Al2O3_ = 9.5 and pH_pzc, TiO2_ = 5.0, the superficial charge density, which can be assumed to be proportional to the difference between particle pH_pzc_ and environment pH, is lower in the case of TiO_2_ particles with respect to Al_2_O_3_ particles. This can be directly related to the amount of bound and free water inside the proton exchange membrane since particles with a higher superficial charge density will be bound to more water molecules. Therefore, for membranes with TiO_2_ particles as filler, more free water will be present inside the solid electrolyte with respect to the amount of free water present inside the membranes with Al_2_O_3_ particles, which have a higher superficial charge density. It is known that water plays a crucial role in the transport of protons across the polymer electrolyte. More specifically, two transport mechanisms are used to describe protons transport in the electrolytes, i.e., Grotthuss and vehicular mechanisms. The former considers the protons’ migration as a “hopping” mechanism in a water chain through the formation of hydrogen bonds between adjacent water molecules [40,41] and it is typically related to high T fuel cells conditions. According to the vehicular mechanism, protons migrate bonded to a “vehicle”, such as water molecules [42,43], and therefore protons’ movement is directly related to the amount of free water inside the membrane. For this reason, this mechanism is typically related to low-T fuel cells conditions. Therefore, free water is essential for the vehicular mechanism to have efficient proton migration. Since DMFC studied in this work worked at 50 °C, i.e., low-T conditions, the proton migration can be associated with the vehicular mechanism and to the presence of free water inside the polymer electrolyte. For this reason, membranes with TiO_2_ particles as filler worked better in these particular operating conditions, leading to the highest measured cell performances.

**Figure 10:**
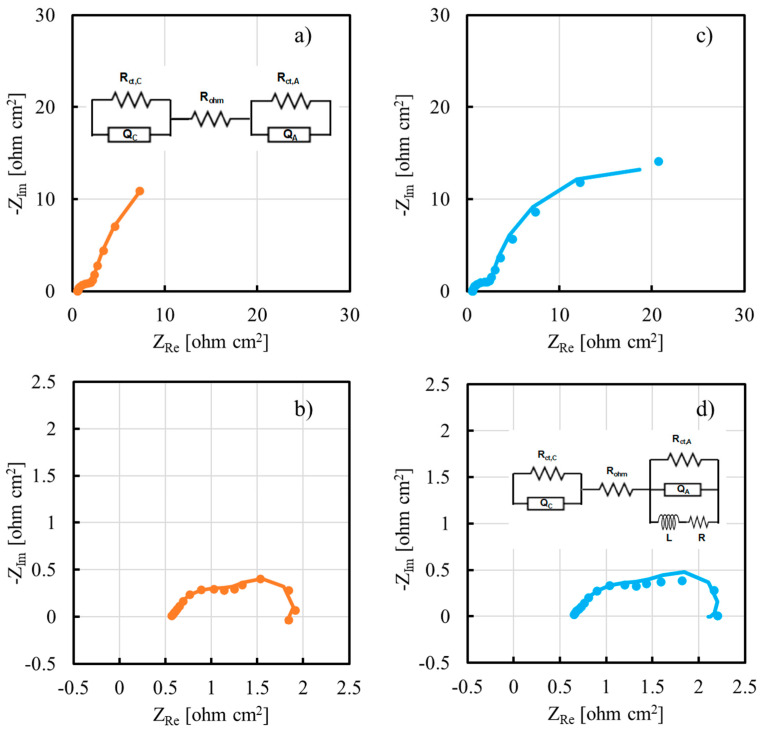
EIS spectra recorded at 50 °C for cells employing TiO_2_ (5 wt%)-containing membranes at (**a**) 550 mV and (**b**) 250 mV. EIS spectra recorded at 50 °C for cells employing Al_2_O_3_ (2% wt)-containing membranes at (**c**) 550 mV and (**d**) 250 mV. Continuous lines: fitting lines. Inset: equivalent electrical circuits.

To have more insight about cell performance as a function of inorganic filler, we recorded EIS spectra at 550 mV and at 250 mV, i.e., in activation region and in ohmic region of the polarization curve, respectively. EIS spectra for cell employing TiO_2_ (5 wt%)-containing and Al_2_O_3_ (2% wt)-containing membranes are reported in Figure 10 in Nyquist representation.

To model the electrochemical behavior of the DMFC, two different equivalent electrical circuits (EECs) were used. EEC used to fit impedance data recorded in activation region, i.e., at 550 mV as cell voltage, comprised a resistance, R_ohm_, which is representative of the cell ohmic contributions, e.g., contact and membrane resistances. This is in series with two parallels (RQ) between a resistance and a constant phase element, CPE (see inset Figure 10a). The former is representative of the charge transfer resistance, R_ct_, directly related to the anode/cathode reaction kinetics, whilst the latter is inserted to model the non-ideal double layer capacitance of the electrode. Two parallel (RQ) are expressed in Nyquist representation by two depressed semicircles, as those shown in Figure 10a,c, suggesting a contribution to the overall impedance of the charge transfer resistance of both half-cell reactions, i.e., methanol oxidation (MOR) and oxygen reduction (ORR), regardless of the inorganic filler used inside the proton exchange membrane. Another EEC was used to fit impedance data recorded in ohmic region, i.e., at 250 mV as cell voltage. In this case, in addition to the previous EEC, a series between a resistance and an inductance was used in parallel to the (RQ) representative of the PtRu anode (see inset Figure 10d). This change in EEC was necessary because of the presence in EIS spectra shown in Figure 10b,d of the beginning of an inductive loop in the low frequencies range. This behavior is usually related to reactions whose kinetics depend on surface coverage, for instance methanol electrooxidation, where the adsorption and desorption of CO species at the electrode play a key role. EIS spectra fitting parameters are reported in Table 1.

Regarding the impedance spectra recorded in the activation region, anode charge transfer resistance, R_ct,A_, is always at least one order of magnitude higher with respect to cathode charge transfer resistance, R_ct,C_, regardless of the nature of inorganic filler. This is due to the methanol electrooxidation reaction which is intrinsically more sluggish than oxygen reduction reaction. It is noteworthy to mention that R_ct,A_ in the case of inorganic filler-modified membrane is always lower with respect to R_ct,A_ estimated with pristine CS/PTA membrane. Since it is difficult to think to any catalytic effect of TiO_2_ or Al_2_O_3_ particles on the MOR reaction, this result is supposed to be due to a better contact between PtRu catalyst layer and the proton exchange membrane.

Regarding the impedance spectra recorded in the ohmic region, R_ct,A_ and R_ct,C_ values are comparable. This result can be explained by considering that, at 250 mV, both electrode reactions have enough overpotential to be fully activated and, therefore, voltage drop is essentially due to the ohmic losses. To understand the reason why it is important to insert an inductance in the EEC to model the electrochemical behavior of the DMFC depending on the impedance spectrum cell voltage, we can consider the MOR as a two-step reaction, based on the following steps [24]:

(6) $${\mathrm{CH}}_{3}\mathrm{OH}\;\overset{k_{1}}{\to }\;{\mathrm{CO}}_{\mathrm{ads}}+4H^{+}+4e^{-}$$

(7) $${\mathrm{CO}}_{\mathrm{ads}}+{\;H}_{2}O\;\overset{k_{2}}{\to }\;{\mathrm{CO}}_{2}+2H^{+}+2e^{-}$$

with *k*_1_ and *k*_2_ the rate constants of the two steps considered for the overall MOR. In particular, Reaction (6) is the methanol oxidation, involving four moles of electrons, which leads to the adsorption of intermediate CO species, whilst Reaction (7) involves the oxidation of CO_ads_ to CO_2_. It can be demonstrated that, if only one intermediate reaction is involved in the overall MOR (i.e., CO_ads_), the admittance, Y (i.e., impedance reciprocal) related to methanol oxidation is expressed by the following equation [24]:

(8) $$Y=\frac{1}{R_{ct}}+\frac{A}{B+j\omega }$$

that is, corresponding to an EEC that depends on the *A* value. The latter depends on many factors, such as the Tafel slopes of Reactions (6) and (7), but also on *k*_1_ and *k*_2_. In particular, if *A* > 0, the suitable EEC to model the behavior of the cell is that reported in the inset of Figure 10d, i.e., EEC with an inductance in series with a resistance. If *A* < 0, the suitable EEC to model the behavior of the cell is that reported in the inset of Figure 10a, i.e., the typical EEC describing two electrodes within in series an ohmic resistance. In the activation region, electrode kinetics control overall cell performance, and in particular MOR is the reaction with highest overpotential, as also demonstrated by the values of R_ct,A_ and R_ct,C_ reported in Table 1. In these cell operating conditions, probably reaction 6a is the rate determining step of the overall MOR, since it involves the exchange of 4 moles of electrons, and therefore *k*_1_ < *k*_2_ and *A* < 0. In the ohmic region, where MOR overpotential becomes high, *k*_1_ and *k*_2_ could have comparable values; therefore, *A* > 0 and it is necessary to insert an inductance in the EEC (see inset of Figure 10d) to suitably model the electrochemical behavior of the cell.

## 4. Conclusions

Hybrid inorganic-organic CS/PTA membranes were successfully synthesized by ionotropic gelation process with the addition of TiO_2_ and Al_2_O_3_ particles in different weight ratios (2 and 5 wt%). The addition of inorganic fillers to pristine CS/PTA membranes led to changes in several properties of the membranes that have a key role in DMFC performance. In fact, TiO_2_-containing membranes were more crystalline with lower liquid uptake and methanol permeability. Al_2_O_3_-containing membranes followed the same trend but resulted to be amorphous and, generally, showed higher liquid uptake and methanol permeability than TiO_2_-containing membranes.

In-cell testing and EIS measurements showed that TiO_2_-containing and Al_2_O_3_-containing membranes can be efficiently used as proton exchange membranes for acidic DMFCs, operating in a single module of 1 cm^2^ as active area, operating at 50 °C fed with 2 M methanol aqueous solution at the anode and oxygen at the cathode. Highest performance was reached by using a membrane with TiO_2_ (5 wt.%) particles, i.e., a power density of 40 mW cm^−2^, almost doubling the performance reached by using pristine CS/PTA membrane (i.e., 24 mW cm^−2^).

These hybrid inorganic-organic membranes, produced by ionotropic gelation process, were found to be suitable for energy storage and conversion applications but they could be also used for other applications, such as electrodialysis, nanofiltration and ultrafiltration, widening the potential application field of this environmentally sustainable membrane production route.

## Figures and Tables

**Table 1:** Fitting parameters of EIS spectra recorded for all the investigated membranes. EECs: inset of Figure 10a,d.

	Pristine CS/PTA		TiO_2_ (2% wt)		TiO_2_ (5% wt)		Al_2_O_3_ (2% wt)		Al_2_O_3_ (5% wt)	
	550 mV	250 mV	550 mV	250 mV	550 mV	250 mV	550 mV	250 mV	550 mV	250 mV
R_ohm_ [Ω·cm^2^]	0.5	0.5	0.7	0.6	0.6	0.6	0.6	0.7	0.5	0.6
R_ct,C_ [Ω·cm^2^]	3.5	1.3	2.8	1.2	1.6	0.7	2.0	0.9	5.7	1.5
Q_C_ [S·s^n^·cm^−2^]	0.04	0.03	0.05	0.13	0.05	0.06	0.04	0.06	0.04	0.07
n_C_	0.79	0.82	0.81	0.56	0.85	0.79	0.88	0.74	0.90	0.75
R_ct,A_ [Ω·cm^2^]	74	1.9	34	2.0	40	1.1	30	1.4	25	1.7
Q_A_ [S·s^n^·cm^−2^]	0.28	0.27	0.29	0.58	0.33	0.41	0.39	0.32	0.38	0.84
n_A_	0.91	1	0.93	0.84	0.91	1	0.92	1	1	0.84
L [H·cm^2^]	-	1.2	-	1.0	-	0.8	-	0.7	-	0.6
R [Ω·cm^2^]	-	1 × 10^−7^	-	2.1	-	1 × 10^−2^	-	1 × 10^−7^	-	0.4
𝒳^2^	3 × 10^−3^	7 × 10^−4^	4 × 10^−3^	3 × 10^−3^	4 × 10^−4^	9 × 10^−4^	2 × 10^−3^	2 × 10^−3^	6 × 10^−3^	8 × 10^−4^

## Data Availability

The data presented in this study are available on request from the corresponding author.

## Supplementary Materials

The following supporting information can be downloaded at: https://www.mdpi.com/article/10.3390/membranes13020210/s1. Figure S1: X-ray diffraction patterns related to pristine CS/PTA membrane; Figure S2: X-ray diffraction patterns related to Al_2_O_3_ powder; Figure S3: Polarization and power density curves related to a single module DMFC of 1 cm^2^ as active area, employing Nafion^®^ 212, operating at 50 °C fed with 2 M methanol aqueous solution at the anode and oxygen at the cathode; Figure S4: Polarization and power density curves related to a single module DMFC of 1 cm^2^ as active area, employing CS/PTA with TiO_2_ (5%) as electrolyte, operating at 50 °C fed with 2 M methanol aqueous solution at the anode and oxygen at the cathode, recorded after 9 h of potentiostatic stability test.
