# Supplementary material for: Effect of TiO2 and Al2O3 Addition on the Performance of Chitosan/Phosphotungstic Composite Membranes for Direct Methanol Fuel Cells
Source: Membranes (Basel). 2023 Feb 8;13(2):210. doi: 10.3390/membranes13020210 (PMC9964683; doi:10.3390/membranes13020210)
Supplement: Supplementary file 1 [file membranes-13-00210-s001.zip › membranes-2187976-supplementary.docx]

**Supplementary Material**

**Effect of TiO_2_ and Al_2_O_3_ addition on the performance of Chitosan/Phosphotungstic composite membranes for Direct Methanol Fuel Cells**

Andrea Zaffora^1,*^, Elena Giordano^2^, Valentina Maria Volanti^2^, Leonardo Iannucci^2^, Sabrina Grassini^2^, Irene Gatto^3^, Monica Santamaria^1^

^1^Dipartimento di Ingegneria, Università degli Studi di Palermo, Viale delle Scienze, Ed. 6, 90128 Palermo, Italy

^2^Dipartimento di Scienza Applicata e Tecnologia, Politecnico di Torino, Corso Duca degli Abruzzi 24, 10129 Torino, Italy

^3^Istituto di Tecnologie Avanzate per l’Energia “Nicola Giordano”(ITAE), Consiglio Nazionale delle Ricerche (CNR), Via Salita S. Lucia sopra Contesse 5, Messina, 98126, Italy

***** Correspondence: andrea.zaffora@unipa.it

**Figure S1.** X-ray diffraction patterns related to pristine CS/PTA membrane.

**Figure S2.** X-ray diffraction patterns related to Al_2_O_3_ powder.


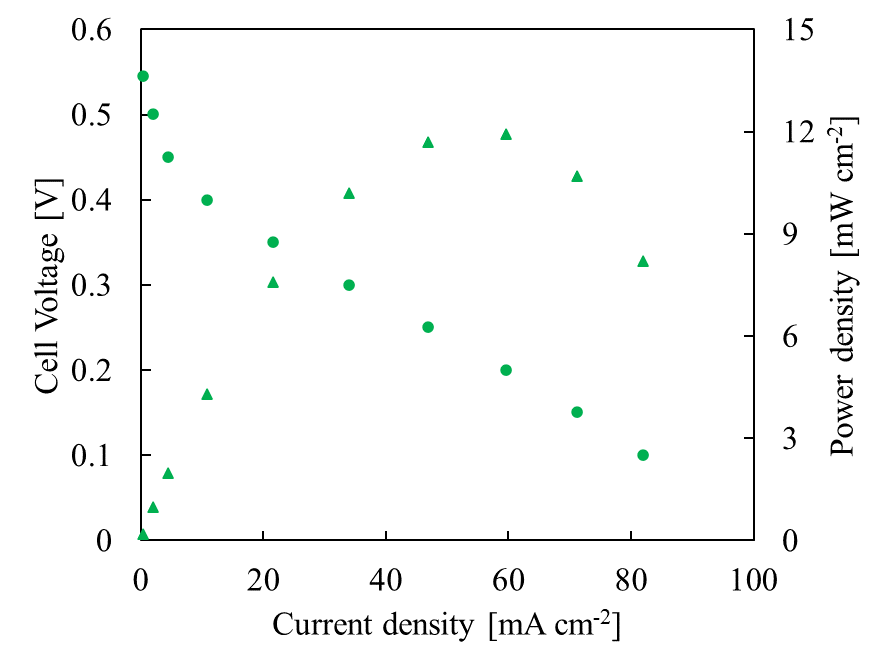


**Figure S3.** Polarization and power density curves related to a single module DMFC of 1 cm^2^ as active area, employing Nafion® 212, operating at 50 °C fed with 2 M methanol aqueous solution at the anode and oxygen at the cathode.


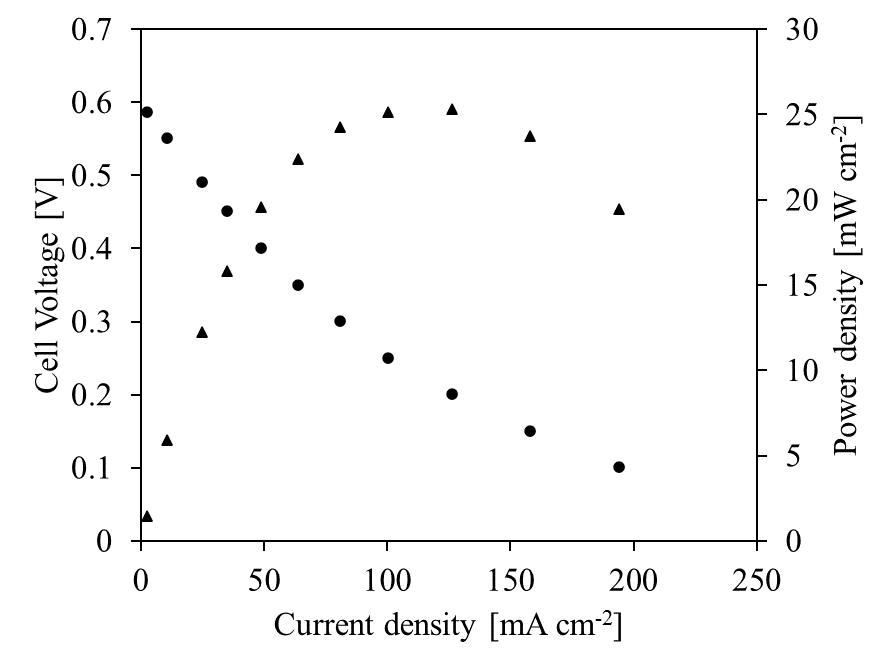


**Figure S4.** Polarization and power density curves related to a single module DMFC of 1 cm^2^ as active area, employing CS/PTA with TiO_2_ (5%) as electrolyte, operating at 50 °C fed with 2 M methanol aqueous solution at the anode and oxygen at the cathode, recorded after 9 h of potentiostatic stability test.
